# Student Behavior during a School Closure Caused by Pandemic Influenza A/H1N1

**DOI:** 10.1371/journal.pone.0010425

**Published:** 2010-05-05

**Authors:** Joel C. Miller, Leon Danon, Justin J. O'Hagan, Edward Goldstein, Martin Lajous, Marc Lipsitch

**Affiliations:** 1 Fogarty International Center, National Institutes of Health, Bethesda, Maryland, United States of America; 2 Department of Epidemiology, Harvard School of Public Health, Boston, Massachusetts, United States of America; 3 Department of Biological Sciences, University of Warwick, Coventry, United Kingdom; 4 Center for Population Health Research, National Institute of Public Health, Cuernavaca, Morelos, Mexico; 5 Department of Immunology and Infectious Diseases, Harvard School of Public Health, Boston, Massachusetts, United States of America; Yale University, United States of America

## Abstract

**Background:**

Many schools were temporarily closed in response to outbreaks of the recently emerged pandemic influenza A/H1N1 virus. The effectiveness of closing schools to reduce transmission depends largely on student/family behavior during the closure. We sought to improve our understanding of these behaviors.

**Methodology/Principal Findings:**

To characterize this behavior, we surveyed students in grades 9–12 and parents of students in grades 5–8 about student activities during a weeklong closure of a school during the first months after the disease emerged. We found significant interaction with the community and other students–though less interaction with other students than during school–with the level of interaction increasing with grade.

**Conclusions:**

Our results are useful for the future design of social distancing policies and to improving the ability of modeling studies to accurately predict their impact.

## Introduction

During the early stages of spread of the pandemic strain of influenza A/H1N1 (H1N1pdm), health officials implemented a number of interventions designed to reduce its spread. One of the most common interventions in the early weeks of the pandemic was temporary school closure, which was applied at levels ranging from individual schools to local school districts to country-wide closures. Among the goals of school closure may be to reduce the number of students infected at the closed school, to reduce transmission from school children to others outside the school, and more generally to slow the spread of infection until other interventions (such as vaccination) are available or until changes in external conditions, such as temperature and humidity, reduce transmission. These goals are not necessarily aligned: a school closure could reduce student-student contacts while enhancing student-community contacts, possibly reducing student infections but accelerating community spread. The specific goal of a closure is a policy decision; how to implement the closure to achieve that goal is a research question. Revised CDC guidelines advise against further school closures for H1N1pdm, but allow for their implementation depending on severity [1]. We did not attempt to evaluate the effectiveness of a school closure, but rather to identify student behaviors that may enhance or detract from the effectiveness.

Previous studies have considered closures affecting all schools across a region either by observing past closures [2], [3], [4], [5] or through mathematical models. The observational studies have seen that school holidays correlate with reductions in influenza-like illness [4] and that a teacher strike in Israel may have reduced respiratory disease in children aged 6–12 [2]. Unfortunately these observations are not directly applicable to disease-induced closures: the closure of a school once many students have been exposed may be less effective than closing the school prior to the disease's introduction. One study of a disease-induced closure in Hong Kong found insufficient data to conclude that the closure was effective [3]. Mathematical models [6], [7], [8] have indicated that disease-induced school closures may help to reduce transmission during an influenza pandemic, but such models require assumptions about student and family behavior during closures. As noted by [9], “simulation studies are only as good as, or at least no better than, the data on which they are based.” Unfortunately, little data exist to inform these studies, so their assumptions range from children remaining at home with a parent caretaker during all school hours (thereby indirectly reducing workplace transmissions) [8] to no change in parents' behavior and increased community interactions for children [7]. Data about the true behavior can be used to calibrate such models and inform future closure policies. To address these data gaps, we investigated a school that was closed due to an outbreak of H1N1pdm shortly after the disease emerged. We surveyed parents and students about what students did during the weeklong school closure. We found that students continued interactions with other students and the community, though with far fewer student-student contacts than would typically occur at school, and the level of interaction increased with grade. Although some parents did stay home from work to care for students, this was not universal.

## Methods

We surveyed students in grades 9–12 and parents of students in grades 5–8 at a school that was closed due to an outbreak of H1N1pdm. We asked about the students' behavior during the closure, their infection status, and their family details. Ethics approval for this study was sought and obtained from the Harvard School of Public Health Office of Human Research Administration. Prior to taking the anonymous survey, parents and students were given a description of the survey and its purpose and were told that the survey was optional. Consent was implied for those who filled in the survey. We did not obtain written consent because that would increase the risk of linking a student with her (or her parents') response.

### Description of the school

We studied Winsor School, a private girls' school in Boston that is divided into two parts: a 176-student “lower school” of grades 5–8 and a 240-student “upper school” of grades 9–12. The two schools share a campus and many facilities, including a common cafeteria (though lunch times do not overlap). About one-third of the students take school buses which are shared between the two schools.

The school was closed for the week from Wednesday 20^th^ through Tuesday 26^th^ May, 2009 inclusive. This period included the Memorial Day holiday (Monday 25^th^ May). The closure resulted from a sudden increase in absenteeism, reaching 48 students on Monday 18^th^ May in grades 5–11 compared with just 4 absences on Monday 19^th^ May, 2008.

### Study Population

All students at the school were eligible to participate.

### Survey Instrument

The survey was distributed in two formats. Parents of lower school students (grades 5–8) were surveyed online through a link emailed to the parents by the school on Thursday 28^th^ May. Upper school students (grades 9–12) were surveyed on paper during a regular school meeting at the beginning of the day on Monday 1^st^ June.

The surveys consisted of 19 multiple-choice questions, taking approximately 10 minutes to complete. The questions addressed symptom history, household details, and activities during the school closure. To keep the survey sufficiently short, we did not ask about normal behavior when schools are not closed as we anticipated that responses for a “typical week” would be unreliable. The surveys were identical for upper and lower school (apart from substitution of “you” for “your daughter” in the upper school survey) except for questions about when fever began in those reporting flu-like symptoms, and the impact the closure had on travel plans. In the first survey (lower school only) we asked about fever onset following 10^th^ May. After learning more about the outbreak, we asked upper school students about the entire month of May. We asked upper school students how their travel plans were altered by the school closure; we did not ask a corresponding question of lower school parents.

### The outbreak

The World Health Organization first announced the existence of H1N1pdm on Friday 24^th^ April. In the ensuing weeks, the disease spread through much of the United States. A letter home from the school on 30^th^ April advised parents that students with fever and respiratory symptoms should stay home “at least 7 days after the onset of illness or until 24 hours after their symptoms resolve, whichever is longer.” Following a substantial increase in absenteeism on Monday 18^th^ May and Tuesday 19^th^ May, the school closed for the week from Wednesday 20^th^ May through Tuesday 26^th^ May inclusive. A new letter home announcing the closure advised that “those in the school community should refrain from all public activities during this time. All students are encouraged to avoid gatherings of Winsor friends or social activities with students from other schools.” Coincidentally with the school closure, on 18^th^ May the death of a school administrator in New York was reported in the local newspaper [10].

Although suspected Boston-area cases were not routinely tested for H1N1pdm, there were confirmed cases in a parent of a student and a student well before the school closure.

## Results

There were 63 parent responses for 176 lower school students (36%) and 188 student responses for 240 upper school students (78%). By grade the response rate was 5/28 (5^th^), 11/40 (6^th^), 12/58 (7^th^), 21/60 (8^th^) with 14 additional lower school responses not reporting grade, and 58/60 (9^th^), 50/60 (10^th^), 31/60 (11^th^), 46/60 (12^th^) with three additional upper school responses not reporting grade.

Given the low response rate of lower school parents, we believe that the lower school sample may be biased towards children who experienced symptoms or parents who were more concerned. Given the variation in illness rates by grade, quantitative comparisons between grades were difficult. Consequently we primarily used the lower school for qualitative statements and the upper school for quantitative study.

The upper school meeting at which the surveys were distributed was not mandatory for grades 11 and 12, so not all received the survey. However, this is unlikely to cause a bias based on infection history.

### Absenteeism and symptoms

**Figure 1:**
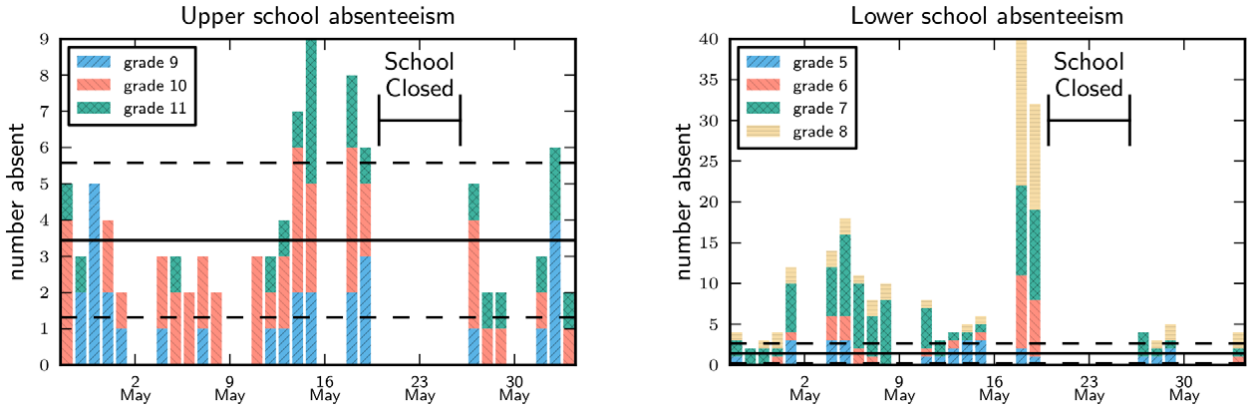
Absenteeism by grade in upper and lower school before and after the school closure. Note different vertical axes. The lower school had significantly higher absenteeism. The mean and standard deviation about that mean for the same period of the previous year is shown in solid and dotted lines respectively.

Aggregate numbers of absences by grade are shown in Figure 1, except for grade 12, which was involved in an independent study project so those students were not generally present at school. On Monday 18^th^ May, the number of absences increased sharply, with highest absenteeism in the lower school. Tuesday experienced similar levels.

**Figure 2:**
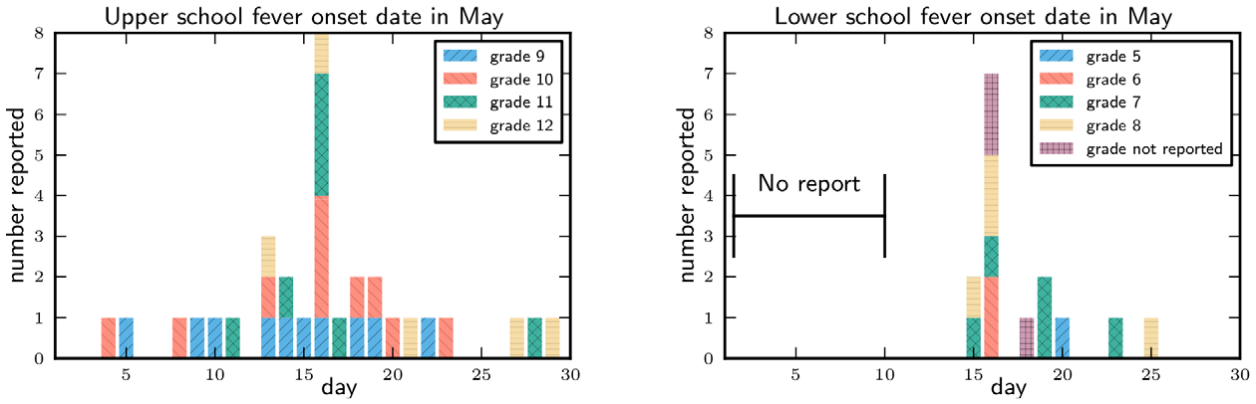
Reported fever onset date of students with ILI. For the lower school we did not ask about days prior to 10^th^ May. The trends appear similar: 16^th^ May has much higher fever onset than any other day for both schools. The absolute number of students reporting onset cannot be reliably compared across the schools (or even between grades) because of different response rates and grade sizes.

In the weeks leading up to the closure, the lower school had elevated absenteeism compared to 2008, particularly in grade 7. In the week before the closure, upper school absenteeism increased, but it decreased on 18^th^ May. Although the absentee trends were different in the two schools, the reported fever onset dates for students with Influenza-Like Illness (ILI), defined as fever with cough/sore throat, do not reflect such a difference. Both schools experienced a sharp peak in fever onset on 16^th^ May (Figure 2).

### Possible Infection Events Prior to Closure

In addition to contacts at school, there were several out-of-school social events that may have contributed to the infections. On Friday 15^th^ May there was a dance for 8^th^ grade students and another social event for many of the 7^th^ grade students. On Saturday 16^th^ May there was a Bat Mitzvah attended by all but three 7^th^ grade students. However, these social events do not explain the peak in absenteeism in grade 6, nor do they explain why the upper school has a similar trend in symptom onset to the lower school, so other less-obvious causes may underlie the infections.

### Upper School Activities During the Closure

We focus on the upper school (grades 9–12) initially because the response rate was higher. In order to study behavior due to the closure rather than behavior modifications caused by infection, we considered just those students who report no ILI. This introduced a risk that the population we studied has different behavior from the population that was infected, but we show below that the behavior of students whose infections occurred long before the closure (and should have been recovered by the time of the closure) was similar to that of those who had no infection. It would be of interest to consider instead the behavior of students who were symptomatic. However, for students symptomatic during the closure, it was not possible with a survey like this to disentangle the effects of closure on their behavior from the effects of their illness on their behavior. Moreover, the goal of school closure is presumably to reduce the mixing between uninfected persons and infectious persons who are feeling well enough to attend school, either because they are not yet symptomatic or because they are mildly symptomatic. The best proxy for the effects of closure on such mixing is arguably the change in behavior of those who are not ill. Finally, the number of students reporting symptom onset immediately before or during the closure is also too small to draw any general conclusion about their behavior.

**Figure 3:**
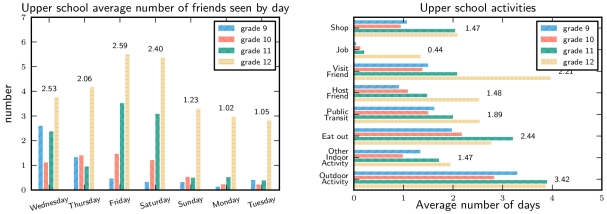
Upper school activity levels by grade. A comparison of the activities of healthy upper school students during the school closure. Activity level in grades 11 and 12 was higher than in grades 9 and 10. Averages are given numerically.

**Figure 4:**
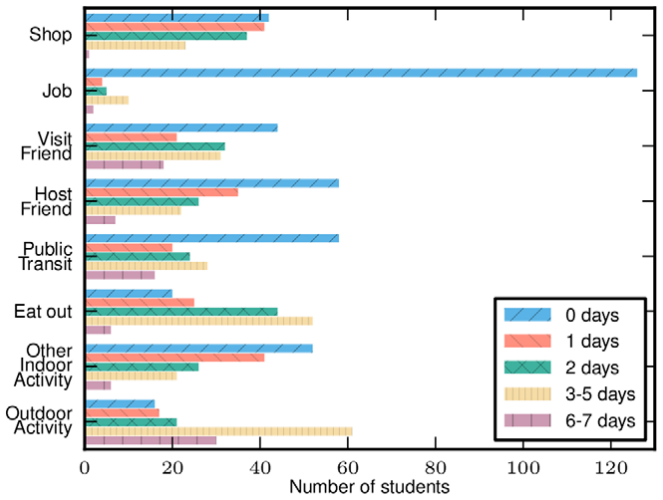
Upper school activity frequency. A comparison of the frequency distribution of different activities among healthy upper school students.

Contact rates of uninfected students at the end of the week were lower than at the beginning (Figure 3a). Contacts substantially increased for grades 11 and 12 on Friday and Saturday. Grade 12 had significantly more contacts than the other grades, particularly late in the week. Because many Grade 12 students were not regularly attending classes at the school prior to the outbreak, they may have felt that they or their friends had not been exposed. In addition to visiting friends, students performed a number of activities in the community (Figure 3b). Each activity that we surveyed (except working at a job) was reported by the majority of students (Figure 4). Participation in most activities was higher in grades 11 and 12 than in grades 9 and 10.

### Comparison of activities by symptom status

**Figure 5:**
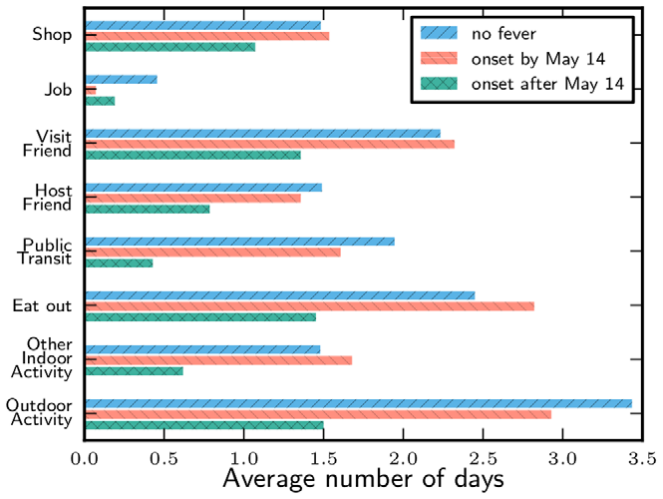
Comparison of the activities of students with different fever onsets. Students are grouped into those who never had symptoms, had fever onset by 14^th^ May, or fever onset 15^th^ May or later. The behavior of students with onset by 14^th^ May is similar to that of students who never became symptomatic.

In Figure 5 we see that those students with earlier infection (by 14^th^ May) had similar behavior during the closure to those who were never infected. Students with early infections were likely to have recovered by the closure. This comparison shows that there is little difference in the behavior of students who were never infected and those who were infected but had recovered, hence there is no evidence to support the theory that students who became sick early were members of a higher risk group. Consequently, using healthy students as a proxy for pre-symptomatic infected students is reasonable. Students reporting fever onset after 14^th^ May reported reduced activity during the closure.

### Duration of absences

Prior to the closure, an email from the school advised students to remain home at least 7 days after symptom onset and 24 hours after symptom resolution. Of 20 students in grades 9–11 who reported ILI onset before the school closure and reported the number of days of class they missed, only 3 did not return to class sooner than 7 days. Those three were infected shortly before the closure, so we cannot be sure if they would have returned sooner had the school not been closed. Four of the twenty students reported missing zero days of school, two of these became symptomatic the day before a school day so were at school the day after becoming symptomatic. This suggests that some of the benefit of school closure seen in the observational studies could simply be a consequence of the fact that symptomatic students are unable to attend school, and so interventions effectively targeting symptomatic students may be able to achieve similar results at reduced social cost.

### Impact on travel

We asked students whether their travel plans changed due to the closure, and if so, whether they increased or decreased their travel. The closure had little impact on the travel plans of respondents. Of 151 students who reported no ILI and answered the question, 116 (77%) reported no change in travel plans, 15 (10%) reported a reduction and 20 (13%) reported an increase. Of 14 students who reported fever by 14^th^ May, 9 (64%) reported no change 1 (7%) reported a reduction and 3 (21%) reported an increase. Of 18 students reporting fever after 14^th^ May, 14 (78%) reported no change, 3 (17%) reported a reduction and 1 (6%) reported an increase. Because of the Memorial Day holiday these results may not be representative of a typical closure.

### Lower School Activities During the Closure

The lower school had a much lower response rate in the survey, and we expect that parents who were more concerned about infections or had symptomatic children are over-represented in the sample. We focus on the responses for those students with no reported illness after 10^th^ May (the cutoff date given in the survey).

**Figure 6:**
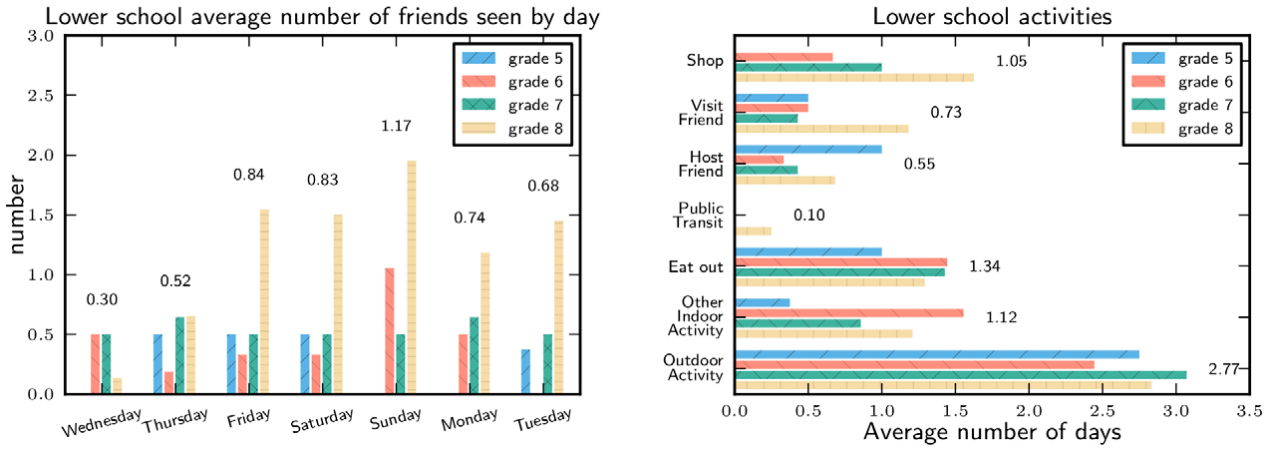
Lower school activity levels by grade. Levels of activity in healthy students are lower than reported by upper school students.

Reported activity rates were significantly lower than in the upper school (Figure 6). This is likely to be because younger students have lower activity, but it may also be because parents had less knowledge of their children's activities.

### Family response

**Figure 7:**
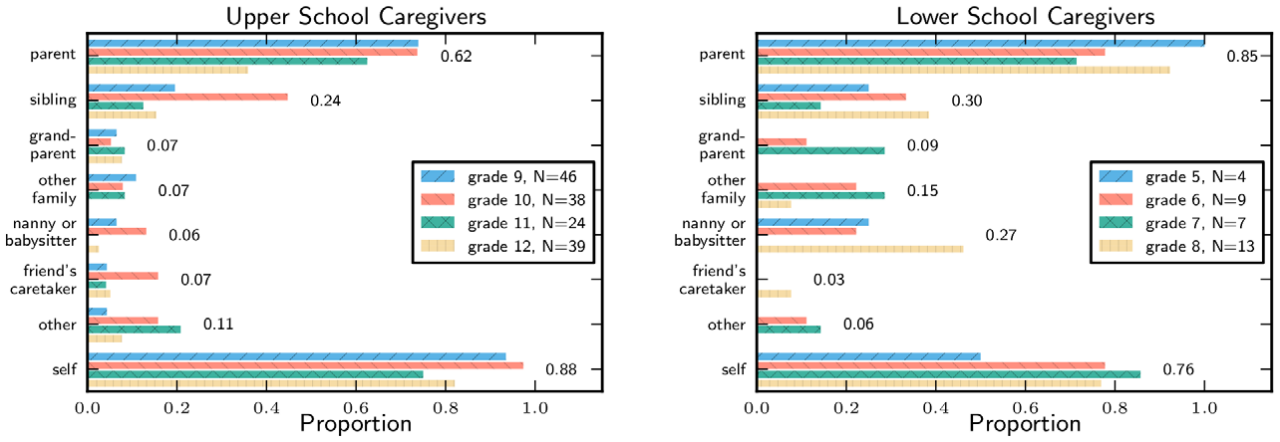
Types of caretakers. The proportion of students with each type of caregiver, restricted to those students with no reported illness.

The main caregivers during the closure were either the students themselves or parents, with no clear trend by grade within each school (Figure 7). Additional caregivers were more common for lower school students. Around 20% of lower school parents report a nanny or babysitter taking care of the student for some of the closure.

**Figure 8:**
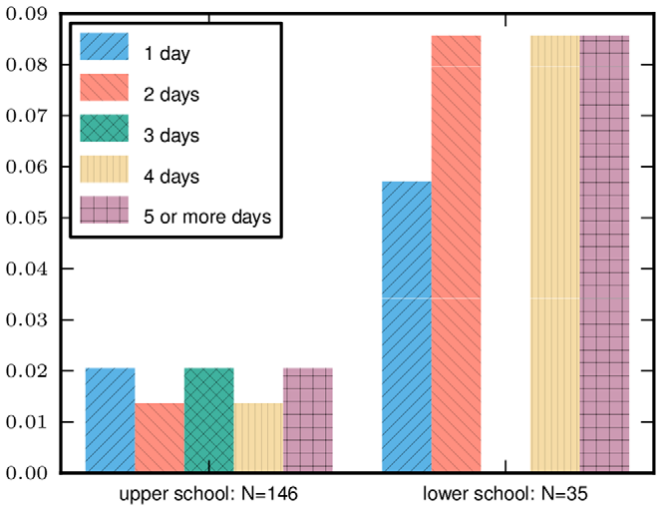
Work missed by caretaker. The fraction of students reporting no ILI whose caretaker stayed home from work for the given number of days. About 9% of healthy upper school students reported a caretaker staying home from work at least one day, while about 30% of healthy lower school parents report a caretaker staying home at least one day.

About 30% of lower school students who had no reported illness had a caregiver remain home from work at least one day. Many had a caretaker stay home for multiple days. In the upper school, the reported proportion was around 9% (Figure 8).

## Discussion

We surveyed parents and students at a school that was closed for one week due to an H1N1pdm outbreak. The results show that students remained active during the closure, with the level of activity increasing with grade, but that the number of contacts with schoolmates was considerably reduced during the closure. Data from behavior surveys cannot directly answer the question of whether transmission between students or to the community was affected by the closure. However, it can help identify those behaviors of students that are likely sources of transmission and help calibrate mathematical models of school closure.

Surprisingly, interaction with other students was lower at the end of the closure than at the beginning, particularly in grades 9–11. We had anticipated that there would be an initial period of fear-based isolation followed by increased contacts as complacency grew. An explanation for the actual observations could be that families were initially unprepared for the closure and students had little to do except visit friends, but as the week progressed families planned additional activities. Alternately, the low level of contacts could represent studying at home for exams that occurred soon after returning to school.

The data suggest students did not closely adhere to advice from the school about behavior to control the spread of infection. Prior to the closure, students were advised to remain home for one week following onset of fever with respiratory symptoms. This was not followed, and some students attended school the day after symptom onset. During the closure, students were advised to avoid contacts with other students and with the community, but our surveys show that they remained active, unless they became symptomatic. Whether it is important for apparently healthy students to avoid social contact for the entire closure is unclear: although direct evidence for or against significant presymptomatic transmission of influenza is weak [11], it is frequently assumed that a significant fraction of infections happen in the presymptomatic stage [12].

Some of the lack of compliance may stem from a misunderstanding of the reasons for or even lack of awareness of the advice. A survey of quarantined individuals in the Toronto SARS outbreak [13] found that only 68% of respondents realized that the quarantine was to prevent them from infecting others. Similarly the instructions may have been interpreted as instructions for protecting individual students rather than protecting others from the students. Instructions that include information about incubation time and infectiousness during incubation time, and the possible consequences for students with pre-existing conditions may achieve better adherence to social distancing measures.

There is limited evidence that social events outside of class may be responsible for a large proportion of the infections. Nevertheless, such unstructured contacts may be more infectious than inclass contacts, so reducing such gatherings may be more cost-effective for preventing transmission than school closure.

This study had a number of limitations. This school closure occurred during a period when very few schools in the Boston area were closed: only 3 of the 135 Boston public schools had a closure period that overlapped. Behaviors during a city- or district-wide closure may differ. Self-reported behaviors may suffer from recall, social-desirability, or other biases. For example, parents may be hesitant to admit to leaving children unsupervised, while students may be hesitant to admit to having a babysitter. Finally, because the closure happened just before the end of the school year, we were unable to survey the students subsequently about how they behaved during a normal week.

A further concern is that the school was a private girls' school, so generalizing to mixed-gender schools or public schools may be inappropriate. However, no single school can provide a representative sample of the demographic details of all schools. Further studies may be needed to better understand the impact on other demographic groups, particularly in populations for which parents will have more limited sick leave. Nevertheless, the current level of knowledge about student behavior is poor to the point that some models assume one parent has no workplace contacts during the closure while others assume that parents' workplace interactions are unchanged. Therefore even rough estimates of student and family behavior allow significant improvements both for modeling and for designing school closure policies.

Future studies are needed to gain a fuller understanding of the impact of school closure. These studies should include questions about what the students/parents understand about the disease, and how that correlates with student activities. Ideally, there should be a control, either as a separate school, or as a survey of the students following a normal school week. This was impractical in the current study because the closure occurred at the end of the academic year.

Our results indicate that a week-long closure of a single school reduces the frequency of contacts between children of school age, but that social interactions and out-of-school activities continue during the closure. These contacts may occur even if parents are advised to keep children out of such interactions.
